# Operando X-ray Raman Ni 2p spectra of LiNi_x_Mn_y_Co1-x-yO_2_ lithium-ion battery electrodes

**DOI:** 10.1557/s43580-025-01397-3

**Published:** 2025-09-25

**Authors:** Adrian Jonas, Selma Erat, Nikolay Ryzhkov, Alexey Rulev, Katja Frenzel, Hongxin Wang, Glennise Faye C. Mejica, Jan Goran T. Tomacruz, Oscar Paredes, Dimosthenis Sokaras, Burkhard Beckhoff, Artur Braun

**Affiliations:** 1 Physikalisch-Technische Bundesanstalt, Abbestr. 2-12, 10587 Berlin, Germany; 2 Laboratory for High Performance Ceramics, Empa. Swiss Federal Laboratories for Materials Science and Technology, Überlandstrasse 129, 8600 Dübendorf, CH, Switzerland; 3 SETI, 339 N Bernardo Ave Suite 200, Mountain View, CA 94043, USA; 4 Department of Medical Services and Techniques, Program of Opticianry, Vocational School of Technical Sciences, Mersin University, 33340 Mersin, Turkey; 5 Department of Nanotechnology and Advanced Materials, Institute of Science, Mersin University, 33340 Mersin, Turkey; 6 Laboratory of Electrochemical Engineering, Department of Chemical Engineering, University of the Philippines, Diliman, 1101 Quezon City, MetroManila, Philippines; 7 SLAC National Accelerator Laboratory, Menlo Park, CA 94025, USA

## Abstract

Lithium manganese nickel oxides are a prominent class of materials used for positively charged lithium-ion batteries. Three different archetype stoichiometries of lithium nickel manganese cobalt oxides (NMC) were studied with bulk-sensitive X-ray Raman and surface-sensitive soft X-ray absorption spectroscopy. We have conducted complementary operando battery X-ray experiments on the NMC622 electrode over one full charge and discharge cycle in a specifically designed operando battery cell. The *ex situ* NEXAFS spectra of the Ni L-edges show distinct differences in their multiplet characteristics depending on the cathode composition. The obtained operando X-ray Raman spectra show changes in the branching ratio of the Ni 2p spectra and confirms that the state of charge of the battery correlates with changes in the bulk of the electrode.

## Introduction

The comprehension and control of electric charge transport properties is essential for the function and integrity of electrochemical energy conversion and storage materials. The transport properties depend on the structure of the materials. We investigate the electronic structure of the class of lithium nickel manganese cobalt oxides (NMC) [1], with X-ray spectroscopy to learn about the ligand-to-metal charge transfer (LMCT). NMCs are the main component of cathodes. We are particularly interested in the metal 2p L-edge multiplet spectra they provide access on the element-specific oxidation state, electronic configuration and local environment. Those can be directly obtained with near-edge X-ray absorption fine structure (NEXAFS) spectroscopy. This method, however, requires soft X-rays with energies ranging from 500 to 1000 eV, which have an information depth of less than one micrometer. Since primary NMC particles may have a size of several micrometers, it is impossible to obtain 2p L-edge spectra of the NMC metal ions from the bulk of the electrode with NEXAFS spectroscopy. This is unfavorable, because the energy of a battery is stored by intercalation of lithium ions in the bulk. By exciting the electrode with hard X-rays in the range of 10 keV, the entire electrode or even battery can be probed. Using a complementary spectroscopic approach, one can extract soft X-ray information from hard X-ray excitation by analyzing the extremely low-intensity X-ray Raman signature [2–5]. This method allows the extraction of 2p spectra even from a battery during operation (operando) [6], provided a specifically designed X-ray spectro-electrochemical cell is used [7].

Kondrakov et al. found that the delithiation process in Ni-rich NMC cathodes can cause structural failure of the electrode [8], related to the charge transfer between the Ni3d and O2p orbitals in the material. Charge transfer is a molecular level process essential for battery operation, which can be elementally assigned by X-ray spectroscopy [9]. Previous studies of LiNiO_2_ have demonstrated the value of using XRS to investigate the electronic structure of Ni-rich layered oxides [10, 11]. The proportionally low concentration of Ni in NMC electrodes poses a particular difficulty in operando XRS.

Here we present our recent work: NMC cathodes of three different compositions (111, 622, and 811) were analyzed with NEXAFS spectroscopy (X-ray absorption) at PTB in Berlin to obtain surface region 2p L-edge reference spectra of Ni. Aliquots of the electrodes were analyzed by *ex situ* X-ray Raman spectroscopy (XRS) at Stanford Synchrotron Radiation Laboratory (SSRL), a division of SLAC National Accelerator Laboratory in Menlo Park, to derive bulk-sensitive 2p L-edge spectra. This *ex situ* comparison was made to test the sensitivity of the new high-q setup of beamline 15–2 at SSRL and to test whether the surface-sensitive NEXAFS and bulk-sensitive XRS show the anticipated similarity. In addition, operando XRS experiments using NMC622 cathodes were performed which provided bulk specific 2p spectra at different states of charge. The aim of the EURAMET Green Deal project OpMetBat: Operando Metrology for Energy Storage Materials [12], is to combine those methods and develop a metrology platform for battery industry. Due to the central role of Ni in the redox reaction of NMC batteries, all studies focused on the 2p L-edge spectra of Ni [13]. All spectra were subject to branching ratio analysis [14] and the Ni L-edge NEXAFS spectra were supported with crystal field multiplet and charge transfer calculations.

## Experimental details

### Electrode fabrication

We prepared two different types of NMC cathodes as described below and in more detail in the Supporting Information file. The NMC622 measured in the XRS operando experiment was prepared by the MEET Battery Research Center in Germany and assembled in a specifically designed X-ray spectro-electrochemical cell [15] equipped with 4 μm thick graphite X-ray windows and cycled at 0.4 mA/cm^2^ using a Biologic SP-300 potentiostat. Additionally, commercially available NMC111, NMC622, and NMC811 electrodes (NANOMYTE^®^, NEI Corporation) were analyzed *ex situ* by NEXAFS and XRS.

### Spectroscopy experiments and computations

The measurements and computational procedures are described in detail in the Supporting Information file.

The X-ray Raman spectroscopy experiments were performed at BL15–2 at SSRL, using a Johann-type spectrometer in Rowland geometry [16]. Measurements of the energy loss scans were performed in the so-called inverse geometry, i.e., fixing the analyzed energy *ω*_2_ while varying the incident energy *ω*_1_. Here, the energy loss spectra were recorded by varying the inicident energy such that the transferred energy *ω* = *ω*_1_ = *ω*_2_ was scanned around the Ni L_2,3_ absorption edges. For all experiments, a high-q spectrometer was employed and operated at the Si(660) reflection corresponding to an analyzed energy of 9.697 keV.

The Ni L-edge NEXAFS data was obtained at the U49/2 PGM beamline at the BESSY II synchrotron radiation facility [17] by the Physikalisch-Technische Bundesanstalt (PTB). The spectra were acquired in inverse partial fluorescence yield mode by detecting the fluorescence radiation of the oxygen K-edge while varying the excitation energy around the Ni L-edge [18].

2p Ni L-edge spectra were calculated for Ni3 + and Ni2 + (Supporting Fig. 2) using CTM4XAS program [19] which was extended from codes that were initially developed for the calculation of atomic spectra by R. D. Cowan et al. [19] to incorporate Butler’s point group symmetry methods for crystal field multiplet calculations by B. T. Thole. The program calculates the spin orbit coupling, Coulomb and exchange terms using the Hartree–Fock method.

**Fig. 1:**
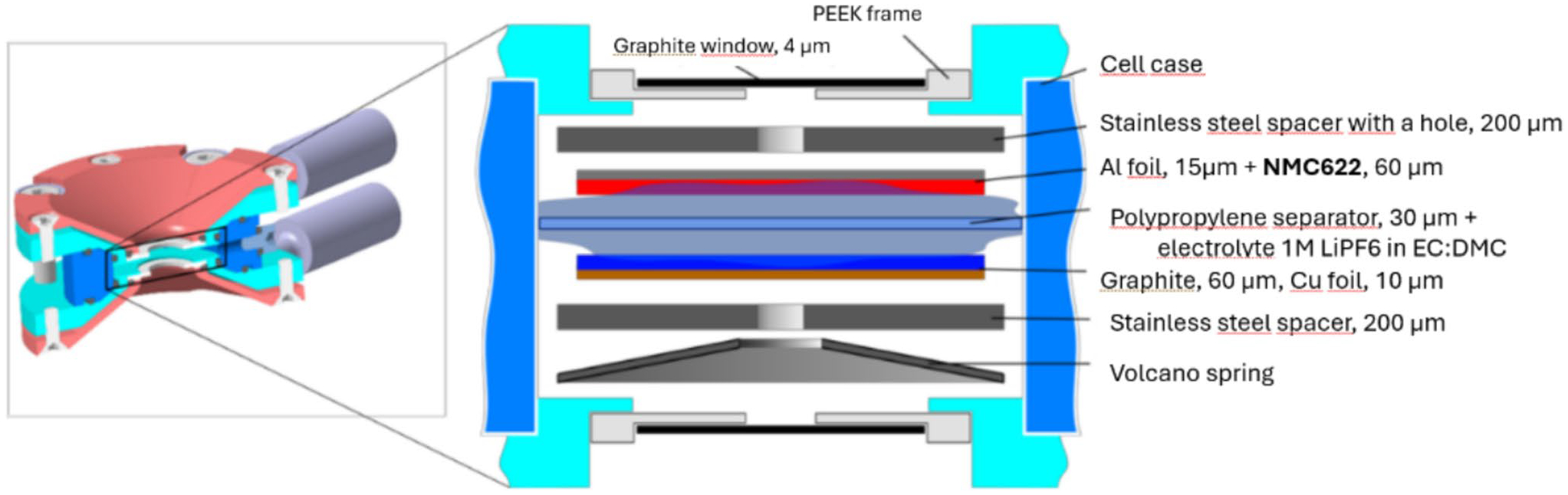
Schematic sketch of the electrode assembly in the X-ray spectro-electrochemical cell indicating the respective thickness of the relevant components. The cell resembles the coin cell 2032 geometry. Note that no modifications the materials have been made

The NMC622 battery was assembled in the operando cell [15] in an argon-filled glove box at the beamline using graphite as the counter electrode (see Electrodes for X-ray Raman at SSRL section), one polypropylene separator, and 1-M LiPF6 in EC:DEC 1:1 electrolyte. The cell resembles the 2032 coin cell geometry. This design, coupled with the fact that no modifications (e.g., holes) were necessary for the active battery materials, means our operando cell provides the best possible experimental representation of actual coin cells. The cell was cycled in the reflection geometry as depicted in Fig. 1. To minimize parasitic X-ray absorption by air and cell components, a 4-μm-thick graphite X-ray window was used (OptiGraph, Berlin), and the optical path between the battery cell and detector was filled by a plastic bag filled with helium gas. The battery cell was operated with a potentiostat type Biologic SP-300 (BioLogic, 38,170 Seyssinet-Pariset, France). Galvanostatic charge/discharge cycling was performed at room temperature using chronopotentiometry within a voltage window of 1.5 V to 4.2 V at a constant current of 400 μA, with the active electrode area being 1 cm^2^ for the positive and negative electrodes, hence a current density of 400 μA/cm^2^.

## Results & discussion

**Fig. 2:**
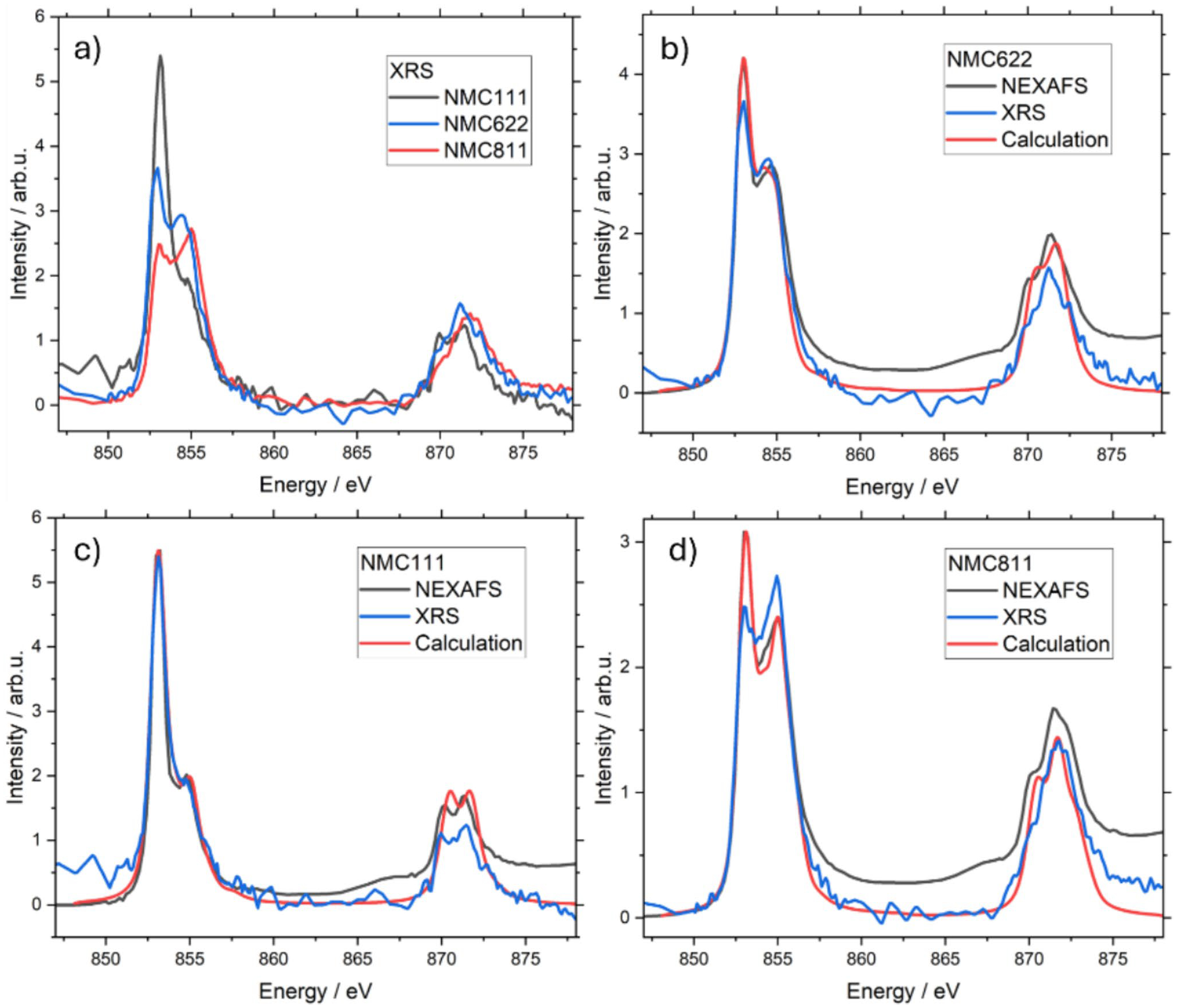
**a** *Ex situ* XRS spectra of all stoichiometries. Comparison of the overlaid experimental NEXAFS, XRS, and theoretical spectra of the 2p Ni L-edges of NMC622 (**b**), NMC111 (**c**), and NMC811 (**d**). The NEXAFS spectra have been normalized and the XRS spectra were energetically shifted to match the NEXAFS spectrum

The 2p Ni L-edges of NMC111, NMC622, and NMC811 investigated *ex situ* are shown in Fig. 2. The normalized spectra of the NEXAFS and XRS measurement are shown together with the theoretical calculations of 2p Ni L-edges of different oxidation states. When comparing the NEXAFS and the XRS spectra, attention has to be paid due to the different methodologies of the techniques [21]. The first feature (853 eV) corresponds to Ni2 + and the second feature (855 eV) corresponds to Ni3 +. This is validated by our calculations (see SI) and the literature [10]. The comparison suggests, that the Ni in all stoichiometries is in a higher oxidation state in the bulk (XRS) than at the surface (NEXAFS) probed by soft X-rays. This is particularly pronounced in the case of NMC811. A possible reason are kinetic limitations during lithiation, since the expected lithiation phase change requires oxygen diffusion which is weaker in the bulk. Similar behavior was observed in previous soft XAS experiments on NMC cathodes [22].

NMC111, NMC622 and NMC622 all show a mixed phase of Ni2 + and Ni3 + configurations. The best agreement between the experimental and theoretical results was achieved for NMC111 showing an 83% Ni2 + and 17% Ni3 + configuration. NMC622 spectra show 64% Ni2 +, 15%Ni 3 +, and 21% Ni3 + L (L denotes an oxygen hole) with charge transfer, whereas NMC811 shows better agreement with 60%Ni2 +, 19% Ni3 +, and 21% Ni3 + L with charge transfer. The charge transfer energy of Δ = 2 eV for Ni3 + calculations was used in both NMC622 and NMC811. The crystal field parameter 10Dq = 3 eV is used for Ni3 + charge transfer calculation for NMC622 and 10Dq = 4 eV for NMC811. The core hole potential U_pd_ and the on-site Coulomb interactions U_dd_ were kept constant (U_pd_ – U_dd_ = 1 eV) for all of the charge transfer calculations. The detailed Ni2 + and Ni3 + calculations (without charge transfer) in steps of 5% as well as the effect of the charge transfer on the Ni2 + and Ni3 + spectra calculations are shown in the Supporting Information.

The NMC622 and NMC811 spectra indicate charge transfer from O 2p to Ni3 + 3d orbitals, known as ligand-to-metal charge transfer. The percentage of Ni3 + configuration is increased for the NMC811 compared to NMC622, while the Ni2 + is decreased, which compensates through charge transfer. The Ni3 + configuration is 3d^3.33^L^4.67^ and 3d^3.8^L^4.2^ in NMC622 and NMC811, respectively. The Ni3 + 3d orbitals are broader compered to Ni2 + 3d orbitals, which indicates stronger hybridization between Ni3 + 3d orbitals and O 2p orbitals. The charge transfer easily occurs within the hybridized orbitals. NMC622 and NMC811 have more Ni atoms (more Ni3 + 3d orbitals hybridized with O 2p orbitals) showing charge transfer, whereas the NMC111 does not show any charge transfer. The NMC622 and NMC811, both shows 21% Ni3 + with charge transfer configuration. Moreover, the amount of the Ni3 + with charge transfer is higher for NMC811 compared to the NMC622. The crystal field effect in the charge transfer calculation is higher in the NMC811 (10Dq = 4 eV) compared to the NMC622 (10Dq = 3 eV) which is attributed to the shorter bond length between O and Ni3 + with charge transfer.

**Fig. 3:**
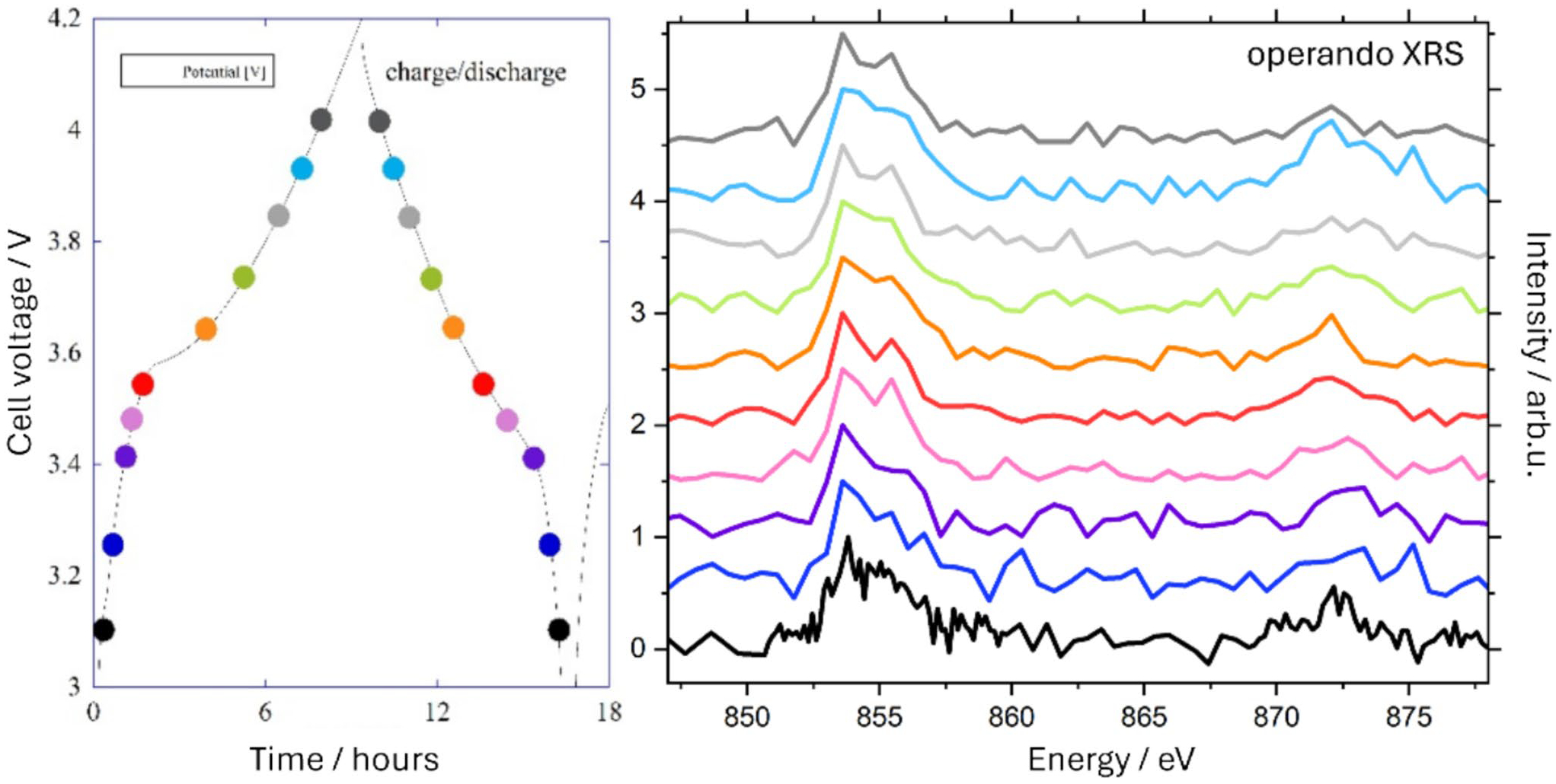
Operando XRS measurement of operando cell 2. The left panel shows the cell voltage with color-coded markers corresponding to the XRS spectra in the right panel. Several spectra at similar cell voltage ranges (charge and discharge) have been averaged to increase the statistics

Three operando cells were built, cycled simultaneously, and measured in series. One of the cells had short circuited during construction. The next cell was measured during the second discharge period and will be referred to as cell 1. The cell could not be recharged after this discharge. The last cell (cell 2) was measured over more than one full cycle. The cell voltage of cell 2 is shown together with the operando XRS spectra in Fig. 3. The cell voltage is shown on the left and the colored markers correspond to the acquired XRS spectra on the right. The charge and discharge processes took about 7 h each. During that time, XRS spectra were measured continuously taking about 25 min each. To improve the statistics, several spectra were averaged over similar cell voltage ranges, regardless of whether they were in the charge or discharge process. The 2p Ni L-edge spectra are showing the Ni mostly in the 3 + oxidation state.

**Fig. 4:**
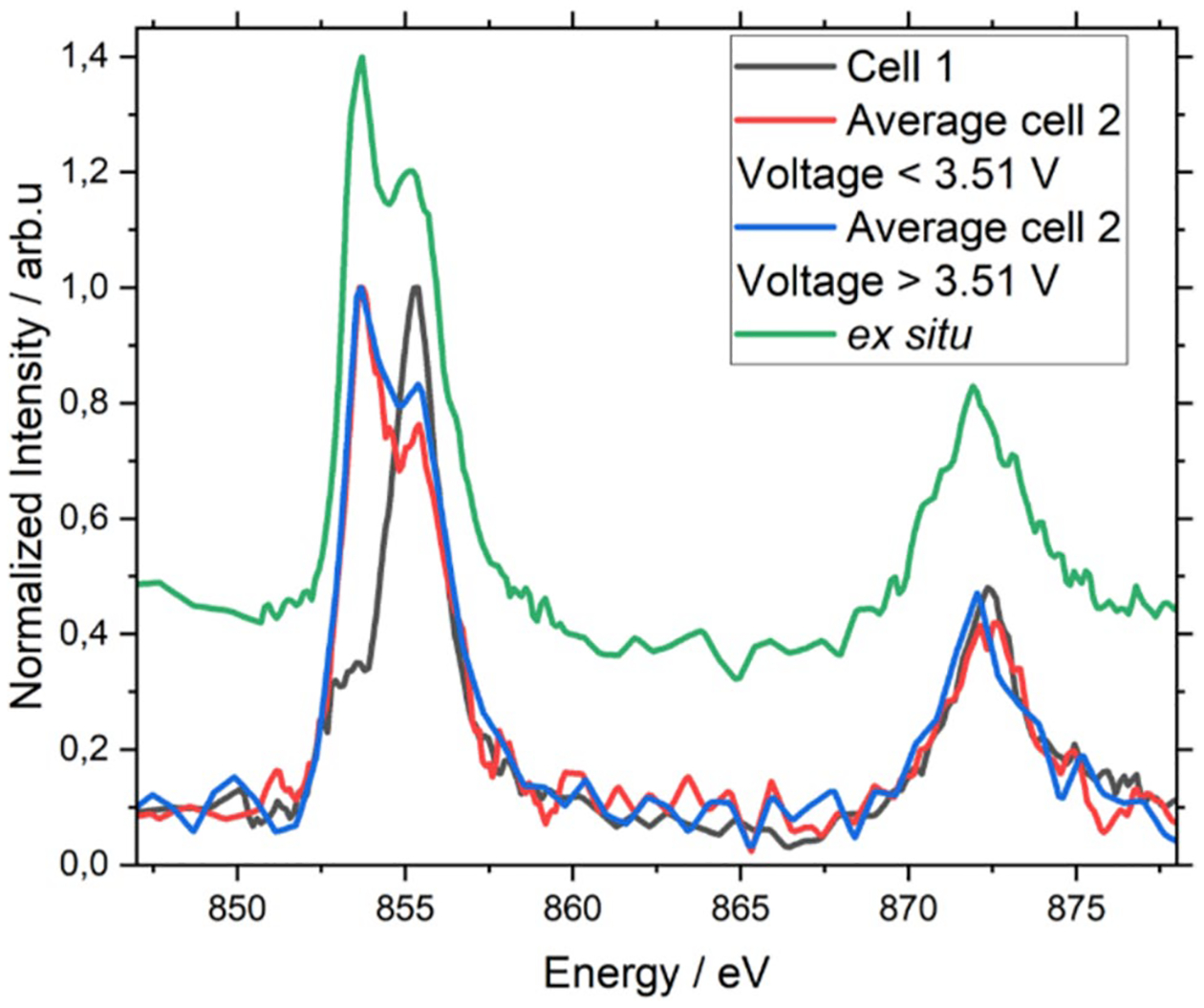
Operando XRS measurement of cell 1 and cell 2. The spectra shown are averaged over a wide range of cell voltages to improve the statistics. For cell 1, all spectra during the discharge process were averaged. For cell 2 the averaged voltage ranges were split into two parts, low voltage and high voltage

Charge dependent differences become more apparent when averaging over even wider voltage ranges. Operando XRS measurements of Cell 1 and Cell 2 are shown in Fig. 4. For comparison, the *ex situ* XRS spectrum of NMC622 is also shown. To improve the statistics, the spectra shown are averaged over a wide range of cell voltages. For cell 1, all spectra of the second discharge were averaged. For cell 2 the measurements were split into two parts, low voltage and high voltage to represent low and high states of charge, respectively. For the higher state of charge the Ni seems to be more predominantly in the 3 + oxidation state in the bulk than for the lower state of charge. The spectrum of cell 1 clearly shows a fully charged (Ni3 +) state of the battery, which was measured throughout the discharge process and did not change. It can be assumed that cell 1 as a whole was functioning. We assume that the area of the sample where the beam hit might have been inactive. Either due to the dose of intense X-ray radiation introduced during the low-efficiency X-ray Raman process or due to the cell window preventing a high and homogeneous cell pressure. When comparing the L3 peaks in cell 2, a higher Ni3 + peak at a higher voltage (higher state of charge) is observed compared to the one of the lower voltages (lower state of charge). Although this is the expected trend for Ni3 + during cycling [23], the Ni2 + peaks did not change. A higher number of batteries is needed to clarify this inconsistency. During each *operando* measurement the background signal of the XRS increased linearly with time. The background mostly consists of Compton scattering [18] and its increase could indicate an influence of the beam toward the sampled area. The formation of gases such as H_2_ or O_2_ caused by electrolyte decomposition could also be a reason, as it can explain the observed swelling of the battery cells windows after the experiment. Although this phenomenon is mostly observed during overcharging [24, 25] or at higher temperatures [26], water contamination [27] or beam effects from harder X-rays [28] might also induce these reactions in Li-ion electrodes and electrolytes.

## Conclusion

In this study, both surface-sensitive NEXAFS and bulk-sensitive XRS were successfully used to investigate the 2p Ni L-edge spectra of NMC622, NMC811, and NMC111 cathode materials. The *ex situ* comparison confirmed that both techniques show similar trends of increasing Ni oxidation state with increasing Ni content, a finding which was validated by theoretical calculations. In particular, bulk-sensitive XRS indicated a higher Ni oxidation state in the bulk compared to the surface, especially for NMC811. Operando XRS measurements provided insight into changes in the bulk electronic structure of NMC 622 during battery cycling, although challenges related to sensitivity and potential sample damage were observed. The analysis of a commercially relevant material such as NMC622 is challenging due to the lower signal sensitivity associated with its moderate nickel content compared to nickel-rich systems. This work demonstrates the ability of hard X-ray excited XRS to probe the bulk electronic structure of battery materials under operating conditions, complementing traditional soft X-ray techniques and providing a valuable tool for understanding complex charge transfer processes.

## Supplementary Material

MRS Advances SI

**Supplementary Information** The online version contains supplementary material available at https://doi.org/10.1557/s43580-025-01397-3.

## Data Availability

Data will be made available upon reasonable request.
