# Supplementary material for: Operando X-ray Raman Ni 2p spectra of LiNixMnyCo1-x-yO2 lithium-ion battery electrodes
Source: MRS Adv. Author manuscript; Available in PMC 2026 Jan 31. (PMC12857916; doi:10.1557/s43580-025-01397-3)
Supplement: MRS Advances SI [file NIHMS2127863-supplement-MRS_Advances_SI.pdf]

MRS Advances

Supporting Information File

Operando X-ray Raman Ni 2p spectra of $LiNi_xMn_yCo_{1-x-y}O_2$ lithium-ion battery electrodes

Adrian Jonas[1], Selma Erat[2,4,5], Nikolay Ryzhkov[2], Alexey Rulev[2], Katja Frenzel[1], Hongxin Wang[3], Glennise Faye C. Mejica[6], Jan Goran T. Tomacruz[6], Oscar Paredes[7], Dimosthenis Sokaras[7], Burkhard Beckhoff[1], Artur Braun[2,*]

[1] *Physikalisch-Technische Bundesanstalt, Abbestr. 2-12, 10587 Berlin, Germany*

[2] *Laboratory for High Performance Ceramics, Empa. Swiss Federal Laboratories for Materials Science and Technology, Überlandstrasse 129, CH - 8600 Dübendorf, Switzerland*

[3] *SETI, 339 N Bernardo Ave Suite 200, Mountain View, CA 94043, United States*

[4] *Mersin University, Vocational School of Technical Sciences, Department of Medical Services and Techniques, Program of Opticianry, 33340, Mersin, Türkiye*

[5]*Mersin University, Department of Nanotechnology and Advanced Materials, Institute of Science, Mersin University, 33340, Mersin, Türkiye*

[6]*Laboratory of Electrochemical Engineering, Department of Chemical Engineering, University of the Philippines Diliman, Quezon City, MetroManila 1101 Philippines*

[7]*SLAC National Accelerator Laboratory, Menlo Park, CA 94025, United States*

Electrode Preparation at MEET

For the X-ray Raman experiment at SSRL, commercial product $LiNi_{0.6}Mn_{0.2}Co_{0.2}O_2$ (NCM622) powder was purchased from BASF by the MEET Battery Research Center at Universität Münster, Germany, and then further processed (mixing, coating, drying, calendaring and cutting) to battery electrodes at the MEET in-house battery production and assembly line described as follows. Deagglomeration and mixing of the electrode pastes was performed by an intensive mixer EL5 from Eirich. For the positive electrode LiNi0.6Mn0.2Co0.2O2 (NMC622, 95 wt.%; BASF, Germany) was used as active material, carbon black (2 wt.%; Super C65, Imerys, France) was used as conductive additive and polyvinylidene difluoride (3 wt.%; PVdF 1100, Kureha, USA) as binder. N-methyl-2-pyrrolidone (NMP, anhydrous, 99.5%, Sigma-Aldrich) was used as solvent at an overall solid content of 76%. The negative electrode consisted of synthetic graphite as active material (Hitachi), carbon black (0.5 wt.%; Super C65, Imerys) a conductive additive, carboxymethylcellulose (CMC, 3 wt.%; Walocel CRT 2000 PA, Chem-point, USA), and styrene-butadiene rubber (SBR, 1.5 wt.%; Lipaton SB 5521, Synthomer, United Kingdom) as binder system. Deionized water was used as solvent at an overall solid content of 42%. Positive electrodes were coated on both sides of a 15 µm Al foil (Nippon Foil, Japan) and negative electrodes were coated on both sides of a 10 µm Cu foil (Schlenk, Germany) using a roll-to-roll "Comma Bar" coating and con-vective drying process (Hohsen HSCM). The single sided areal capacities were set to 2.3 mAh cm^{-2} for the positive electrode (total mass loading of 13.5 mg cm^{-2}; practical capacity of 180 mAh g^{-1}) and to 2.8 mAh cm^{-2} for the negative electrode (total mass loading of 7.9 mg cm−2; theoretical capacity of 372 mAh g-1) resulting in a N/P ratio of 1.2/1. The electrodes were calendared to a porosity of 30% by an GKL400 calender from Saueressig. A polypropylene monolayer was used as separator (Celgard 2500, Celgard, USA) and soaked by a standard electrolyte mixture comprised by 1M LiPF6 in ethylene carbonate and ethyl methyl carbonate (EC:EMC, 3:7 wt.) + 2 wt.% vinylene carbonate (VC).

Electrodes for *ex-situ* NEXAFS at PTB and *ex-situ* X-ray Raman at SSRL

The three compounds subject to investigation were $LiNi_{0.6}Mn_{0.2}Co_{0.2}O_2$ (NMC622), $LiNi_{0.8}Mn_{0.1}Co_{0.1}O_2$ (NMC811), $LiNi_{0.33}Mn_{0.33}Co_{0.33}O_2$ (NMC111). Electrode sheets with these materials were purchased from NEI Corporation (NANOMYTE®, NEI Corporation, Somerset, NJ 08873, USA). For the measurements, pieces of each sample were cut and placed in the ultra-high vacuum (UHV) equipment. The electrodes contain 90% NMC active material, 5% PVDF binder and 5% conductive carbon black. The active material layer is approximately 60 µm thick, coated on a 16 µm thick aluminum foil.

Electrodes and battery cell for *operando* X-ray Raman at SSRL

For the *operando* X-ray Raman experiment at SSRL, we used NMC622 positive electrodes (cathodes) which were produced by the MEET Battery Research Center at Universität Münster, Germany from commercially available $LiNi_{0.6}Mn_{0.2}Co_{0.2}O_2$ (NCM622, BASF), mixed with carbon black and polymer binder, coated on 15 µm aluminium foil. MEET also provided negative electrodes (anodes) from carbon coated on 10 µm copper foil. We used Celgard 2500 as separator and 1M $LiPF_6$ in EC:DEC 1:1 as electrolyte. The X-ray window of the *operando* cell was a 4 µm thick highly oriented pyrolytic graphite foil (Optigraph GmbH, Berlin, Germany). The sandwich structure of the battery cell and further information is shown in the main article Figure 1.

Spectroscopy experiments

NEXAFS at PTB

NEXAFS data was acquired by the Physikalisch-Technische Bundesanstalt (PTB) using the U49/2 PGM beamline at the BESSY II synchrotron radiation facility [14]. Ni $L_{3,2}$-edge NEXAFS spectra were recorded using 118 energy steps with a measurement time of 20 seconds per step using a silicon drift detector. Finer energy steps of 0.2 eV were used around the absorption edge features and larger steps in the pre- and post-edge regions. Nine individual spectra were averaged to improve the signal-to-noise ratio. The inverse partial fluorescence yield mode was used by detecting the fluorescence of the oxygen K-edge fluorescence [15]. The fluorescence intensities were divided by the incoming photon flux I_0, which was measured using a photodiode. The NEXAFS spectra were normalized to 0 (pre-edge) and 1 (post-edge).

X-ray Raman at SSRL

X-ray Raman spectroscopy (XRS) experiments were performed at beamline 15-2 of the Stanford Synchrotron Radiation Lightsource (SSRL), using a Johann-type spectrometer in Rowland geometry [13]. At the end station operates a high-q instrument with 30 analyzer crystals, spherically bent-diced analyzer crystals with 1m bending radius, covering scattering angles between 110° and 160°. Measurements of the energy loss scans were performed in the so-called inverse geometry, i.e., fixing the analyzed energy ω_2 while varying the incident energy ω_1. Hence, the energy-loss spectra were recorded by varying the inicident energy such that the transferred energy $\omega = \omega_1 - \omega_2$ was scanned around the Ni $L_{2,3}$ absorption edges. For these experiments, the high-q spectrometer was employed and operated at the Si(660) reflection corresponding to an analyzed energy of 9.697 keV with an energy resolution of 0.58 eV measured as the full width at half maximum of the elastic line. The measurement time per energy channel was set to 30s. Accurate energy calibration of the energy-loss scans was achieved by periodic measurements of the elastic line. The magnitude of the momentum transfer covered by the spectrometer was between 4.5 a.u. and 5.4 a.u.

Operando X-ray Raman at SSRL

The NMC622 electrode was assembled in the operando cell [12] in an argon filled glove box at the beamline using graphite as counter electrode (see Electrodes for X-ray Raman at SSRL section), polypropylene separator, and 1M $LiPF_6$ in EC:DEC 1:1 electrolyte. A carbon counterelectrode on 10 µm Cu current collector was used. The cell was run in the reflection geometry. To minimize parasitic X-ray absorption by air (N_2, O_2) and cell components, an X-ray graphite window of 4 micrometer thickness was used (OptiGraph, Berlin), and the optical path between cell and detector was breached by a helium gas filled plastic bag. The cell was operated with a potentiostat type Biologic SP-300 (BioLogic, 38170 Seyssinet-Pariset, France). Cells were cycled continuously during the measurement. The measurement time per spectrum was approximately 25 minutes, which gives the lower limit of time resolution of the operando measurment. It became apparent that several spectra had to be averaged due to counting statistics, which further reduces the time resolution. We have used a 4 micrometer thin Highly oriented pyrolytic graphite (HOPG) film as x-ray window in this X-ray Raman experiment, whereas a 380 micrometer thick beryllium plate was used in a previous X-ray Raman experiment [5]. The X-ray transmission at 10 keV is over 99% for HOPG (4 micrometer thickness). At 800 eV it is only 2%. For Beryllium 380micron, it is 96% at 10 keV and 5E-26 at 800 eV. However, the absorption in the soft x-ray range is here not relevant because the soft x-ray information is obtained at high x-ray energies.

Results and discussion

The dipole allowed transtion from inital state to final state is following: the ($2p^6 3d^n \rightarrow 2p^5 3d^{n+1}$) transition results in a 2p core configuration that splits into two states at different energy due to spin-orbit coupling. The L_3 and L_2 edges further split into two states due to crystal field effect. The calculations were performed for octahedral symmetry, Oh, reducing the Slater integrals 65% and for Ni2+ and 50% for Ni3+ along with the crystal field splitting parameter, 10Dq = 3 eV. The Lorentzian broading is used as 0.3 and 0.4 for the higher energies (≥870 eV) and Gaussian broading is used as 0.3. The experimental 2tg peaks of L3 edges reagrding peak positions and peak intensity were used as references during the comparision.

The best agreement was successfully obtained for NMC111 showing 83% Ni2+ and 17% Ni3+ configurations. The NMC622 spectra shows 64% Ni2+, 15%Ni 3+ and 21% Ni3+$\underline{L}$ ($\underline{L}$ denotes Oxygen hole) with charge transfer whereas NMC811

shows better agreement with 60% Ni2+, 19% Ni3+ and 21% Ni3+L with charge transfer. The charge transfer energy Δ=2 eV for Ni3+ calculations both for the NMC622 and NMC811. The crystal field parameter 10Dq = 3 eV is used for Ni3+ charge transfer calculation for the NMC622 and 10Dq = 4 eV for the NMC811. The core hole potential Upd and the on-site Coulomb interactions Udd were kept constant (Upd – U_{dd} = 1 eV) for all of the charge transfer calculations. The detailed Ni2+ and Ni3+ calculations (without charge transfer) in step of 5% is shown in Supporting Materials. The effect of the charge transfer on the Ni2+ and Ni3+ spectra is shown in the Supporting Materials. The charge transfer energy and the crystal field effect 10Dq are 2 eV and 3 eV, respectively.

Table S1: Peak heights and centroid positions of the 2p derivced from XRS.

	NMC111	NMC622	NMC811	Cell 1 Average	Cell 2 low V	Cell 2 high V
β	0.612	0.779	0.689	0.69	0.72	0.7

One common metric for the quantitative assessment of the NEXAFS spectra is the branching ratio β, this is, the ratio of the L_3 intensity versus the intensity of L_3+L_2,

$$\beta = \frac{L_3}{L_3 + L_2}$$

The such obtained branching ratios are β=5/8 for NMC811, 2/3 for NMC622, and 3/4 for NMC111. These values in addition to the calculations also indicate that NMC811 and NMC622 have the Ni in the low spin state Ni3+, whereas the large branching ratio of NMC111 indicates high spin Ni2+ or even high spin Ni1+.

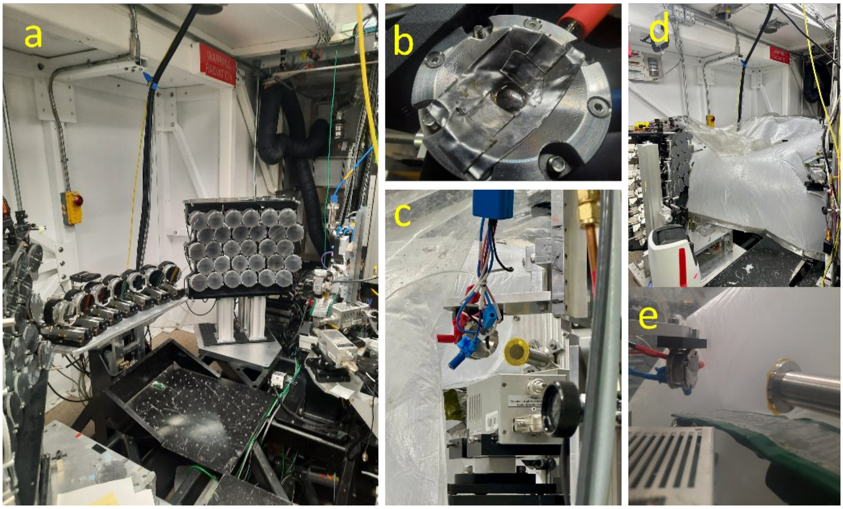

Supporting Figure 1: Setup of beamline 15-2 at SSRL. (a) 55 analyzer Si crystals collecting the photons emitted by the sample upon irradtion with X-rays. Sample position is close to the middle right side of the photo. (b) Photo of the front side of the operando cell with the HOPG x-ray window in the middle. (c) Operando cell, connected to blue and red clamps and cables. On the right side from the cell is the X-ray beam tube, closed with a Kapton(R) foil. Optical path and sample normal enclose a 45° angle towards the azimuthal crystal array on the left. (d) Optical path between crystal array and battery is bridged by a plastic bag filled with helium gas. (e) On the right side the X-ray beam tube, pointing at 45° degree to the operando cell surface on the left side.

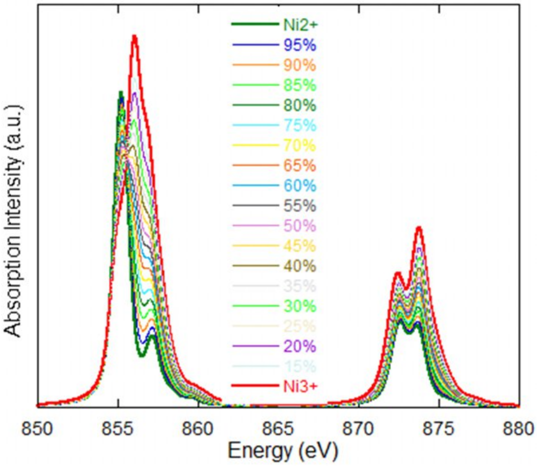

Supporting Figure 2: Calculated Ni2+ and Ni3+ spectra and linear combination of Ni2+ and Ni3+ in step of 5%. The written percentage in the figure corresponds the percentage of Ni2+ as taking into account (Ni2+) % + (Ni3+)% =100%
